# Beyond form and functioning: Understanding how contextual factors influence village health committees in northern India

**DOI:** 10.1371/journal.pone.0182982

**Published:** 2017-08-24

**Authors:** Kerry Scott, Asha S. George, Steven A. Harvey, Shinjini Mondal, Gupteswar Patel, Rajani Ved, Surekha Garimella, Kabir Sheikh

**Affiliations:** 1 Department of International Health, Johns Hopkins School of Public Health, Baltimore, Maryland, United States of America; 2 Public Health Foundation of India, New Delhi, Delhi National Capital Territory, India; 3 University of the Western Cape, Cape Town, Western Cape, South Africa; 4 The University of Newcastle, Callaghan, New South Wales, Australia; 5 National Health Systems Resource Centre, New Delhi, Delhi National Capital Territory, India; Post Graduate Institute of Medical Education and Research School of Public Health, INDIA

## Abstract

Health committees are a common strategy to foster community participation in health. Efforts to strengthen committees often focus on technical inputs to improve committee form (e.g. representative membership) and functioning (e.g. meeting procedures). However, porous and interconnected contextual spheres also mediate committee effectiveness. Using a framework for contextual analysis, we explored the contextual features that facilitated or hindered Village Health, Sanitation and Nutrition Committee (VHSNC) functionality in rural north India. We conducted interviews (n = 74), focus groups (n = 18) and observation over 1.5 years. Thematic content analysis enabled the identification and grouping of themes, and detailed exploration of sub-themes. While the intervention succeeded in strengthening committee form and functioning, participant accounts illuminated the different ways in which contextual influences impinged on VHSNC efficacy. Women and marginalized groups navigated social hierarchies that curtailed their ability to assert themselves in the presence of men and powerful local families. These dynamics were not static and unchanging, illustrated by pre-existing cross-caste problem solving, and the committee’s creation of opportunities for the careful violation of social norms. Resource and capacity deficits in government services limited opportunities to build relationships between health system actors and committee members and engendered mistrust of government institutions. Fragmented administrative accountability left committee members bearing responsibility for improving local health without access to stakeholders who could support or respond to their efforts. The committee’s narrow authority was at odds with widespread community needs, and committee members struggled to involve diverse government services across the health, sanitation, and nutrition sectors. Multiple parallel systems (political decentralization, media and other village groups) presented opportunities to create more enabling VHSNC contexts, although the potential to harness these opportunities was largely unmet. This study highlights the urgent need for supportive contexts in which people can not only participate in health committees, but also access the power and resources needed to bring about actual improvements to their health and wellbeing.

## Background

Health committees in low- and middle income countries play an important role in health systems. Their contributions can include improved management and accountability of peripheral health services, participatory health planning and local resource mobilization, expanded community support for health workers, as well as improved reach of health services and health messages [1–7].

Despite their prevalence and documented effectiveness [7], health committees are permeable and interconnected to contextual factors that impinge their performance in numerous ways [7,8]. Committees have been hampered by a lack of formal mandate and authority [9], at times linked to broader bureaucratic resistance to decentralization [10–12]. The functionality of the broader health system has a major effect on how health committees operate; in weak health systems, health staff cannot respond to committee demands and health committees can develop strained relations with health center staff [1]. Unjust social structures are often replicated in health committees, with some failing to include women [13] or other marginalized social groups [5,14].

Studying context is increasingly recognized as a vital aspect of what makes health policy and systems research a dynamic social science subject [15]. Context shapes the success of interventions; the same intervention components will bring about highly variable results across different settings [16–18]. The reasons behind these differences are increasingly being examined using approaches such as realist evaluation, which moves beyond the traditional evaluation question of “does the intervention work?” to “what works, for whom, in what circumstances?” [19]. Such contextual analysis can help improve specific interventions, either by identifying aspects of the environment that must be altered to enable the intervention to succeed, or aspects of the intervention that can be adjusted to better suit the context. In addition, greater understanding of context, and how context interacts with an intervention, can generate transferrable knowledge to inform interventions elsewhere.

Informed by this consideration of context, this present paper undertakes a contextual analysis of village health, sanitation and nutrition committees (VHSNCs) in rural north India. Officially, over 500,000 VHSNCs have been formed since their inception in the mid-2000s [20]. They are mandated to bring together many key actors in the rural health system: community health workers (called Accredited Social Health Activists (ASHAs)), village nutrition and child development workers (called anganwadi workers), Auxiliary Nurse Midwives (ANMs), members of the local elected government (called the gram panchayat), and interested citizens. VHSNCs are to convene monthly meetings, conduct local health planning, and monitor the anganwadi system and government health services. VHSNCs are also to receive a yearly “untied fund” of Rs. 10,000 (US$150) to spend on local health needs as they see fit. Many committees, however, have been found to be largely inactive [21–24].

In 2013, the Ministry of Health and Family Welfare (MoHFW) released new VHSNC guidelines, which clarified VHSNC objectives and activities, recommended membership be expanded from seven to 15 people, and outlined the training, capacity building, and support that VHSNCs should receive. The National Health Systems Resource Center (technical advisor to the MoHFW) collaborated with the Public Health Foundation of India to conduct implementation research on NGO experiences of supporting such VHSNC strengthening. We use pseudonyms for the NGO’s name, the research block, all respondents, and all local locations. As a part of this research, “SEEK,” a small NGO, strengthened VHSNCs in 50 villages in northern India for 1.5 years (2014–2015) by (1) increasing community awareness about the VHSNC; (2) expanding VHSNC membership; (3) training VHSNC members; (4) supporting monthly VHSNC meetings and quarterly cluster-level meetings; and (5) supporting VHSNC activities.

At the end of the study, notable gains were made in terms of form (i.e. membership expanded through a participatory process to 15 people, at least eight members at most meetings, and appropriate representation by gender, caste and religion) and functioning (i.e. most VHSNCs held monthly meetings, identified a wide range of issues that required improvement, and sought to address them largely by appealing to government officials). SEEK was committed and effective at delivering the package of supports (discussed in S1 File). However there was little success in improving local health, sanitation or nutrition: VHSNC requests to government officials (e.g. for filling staffing vacancies, equipping facilities with supplies and medicine, training the untrained ASHAs, improving access to water), generally received no response or resulted in no action; the untied fund did not arrive so there was no local budgeting; and local health planning and monitoring were very limited. To further understand why this was the case, we sought to examine in more detail how VHSNCs navigated their contextual environment and how this may explain the dearth of concrete improvements. Informed by George et al.’s [8] framework, which sees health committees as embedded within a larger dynamic system with multiple interconnecting layers and actors, our paper analyzed VHSNCs as contextually contingent interventions with potential to both influence and be influenced by their broader environment.

## Methods

### Study location

SEEK’s work took place in “Manujpur” block (sub-district level, population approximately 300,000), an impoverished rural area within 250 km of New Delhi. A large portion of the population is especially marginalized: 18% scheduled caste (SC), 12% scheduled tribe (ST) and 20% Muslim. Literacy is 80% for men and 50% for women. People in the region are primarily farmers, the majority with small plots or as migrant laborers, or work in construction and transportation. Houses are generally mud or concrete one-story buildings, with no indoor plumbing and irregular electricity. Stagnant dirty water and piles of garbage are an issue in all the villages, and toilets are uncommon. Many villages are accessible only by poor quality roads that become almost impassable due to mud and flooding in the monsoon season.

### Study design

This in-depth longitudinal qualitative study took place over the course of 1.5 years in 2014–2015 and was guided by case study design [25]. Interventions often fail to bring about expected improvements once implemented on the ground due to a range of factors including ‘real world’ contextual factors [26]. Case study research enables deep exploration of contemporary phenomenon within a real life context [25], making it a valuable method for studying program implementation and capturing the contextual factors that may hinder an intervention’s effectiveness.

While the VHSNC-strengthening intervention was implemented in 50 villages, we focused on four villages as our case study sites, to enable deep understanding of the social, political, and financial dynamics of VHSNC participation and action at the village level. We selected these villages (“Jhorkibas,” “Sojjanpur,” “Shadeeka” and “Hanwari”) purposively [27], to enable us to understand locations that were more and less geographically remote and which had populations that were highly marginalized (primarily Muslim, SC and/or ST) and less marginalized (a greater proportion of the less marginalized other backwards caste (OBC), and general castes).

### Data collection

Data collection involved 74 in-depth interviews (IDIs) and 18 focus group discussions (FGDs) with a range of village- and block-level stakeholders over the 1.5 years (Table 1). Respondents were selected purposively to explore diverse perspectives and experiences (e.g. active and inactive VHSNC members). IDIs enabled us to understand individual perspectives and experiences with VHSNC involvement, while FGDs explored social dynamics and norms among community members, first around general community issues, such as health needs and prior collective engagement, and, as the intervention unfolded, about the VHSNC’s potential and challenges. We also observed 54 key activities, primarily in the four case study villages, including social mobilization by the NGO, VHSNC meetings, VHSNC member trainings, and cluster level meetings.

**Table 1:** Data overview by respondent characteristics.

Respondent type		Gender		
		Male	Female	Total
**Interviews**				
VHSNC members				
	General community	21	11	32
	ASHA	0	8	8
	Anganwadi worker	0	12	12
	Gram panchayat (local government) member	3	2	5
	*Total interviews with VHSNC members*	*24*	*33*	*57*
Non-VHSNC members				
	General community	4	1	5
	ANM	0	3	3
	ASHA supervisor	1	1	2
	Block Chief Medical Officer	1	0	1
	NGO staff	3	3	6
	*Total interviews with non-VHSNC members*	*9*	*8*	*17*
*Total Interviews*		***33***	***41***	***74***
**Focus group discussion**				
VHSNC members		4	8	12
General community, includes some VHSNC members		3	3	6
***Total Focus Groups***		***7***	***11***	***18***

As shown in Table 1, women were overrepresented among our sample. We included additional IDIs and FGDs with women because several key VHSNC and frontline health worker positions (ASHA, anganwadi, ANM) were reserved for women and because male voices tended to dominate public activities such as VHSNC meetings; additional FGDs with women ensured we included sufficient female perspectives. The IDIs sampled Muslims (n = 9), marginalized (i.e. SC and ST) Hindus (n = 33) and non-marginalized (i.e. OBC and general) Hindus (n = 32). FGDs were mixed Hindu and Muslim (n = 6), mixed marginalized and non-marginalized Hindu (n = 4), only marginalized Hindu (n = 5), or only non-marginalized Hindu (n = 3).

Interviews and focus groups were conducted using topic guides (c.f. S2 and S3 Files) and explored the local context, including respondent experiences with government services (particularly the health system and anganwadi system), how communities in the village interact with one another, and earlier collective action. As VHSNCs became more active, the interviews and focus groups focused on respondent experiences participating in the VHSNC, VHSNC activities and functionality, engagement with government, and other factors that facilitated or hindered VHSCN functionality. IDIs and FGDs were audio recorded with the respondents’ permission and were then translated and transcribed from Hindi into English.

The study received ethical approval from the PHFI Institutional Ethics Committee (TRC-IEC-178/13) and the World Health Organization’s Research Ethics Review Committee (RPC581).

### Data analysis

Our analysis was guided by thematic content analysis, whereby we developed a coding frame inductively and deductively, first based on the theoretical interests guiding the research and later modified based on salient issues that arose from the data itself [28]. This coding framework was applied to the transcripts, supported by the qualitative software ATLAS.ti v7, to enable us to group text segments into topics. Data outputs consisting of the text segments for each code were then read and re-read to extract the salient, common and significant basic themes. Basic themes were initially descriptively grouped into the four contextual spheres from the George, et al. [8] framework: community, health facility, administration and society. We then further synthesized the findings into six overarching analytic themes on the dynamic interaction between contextual factors and the health committee

## Findings

We now discuss contextual features arranged by the six overarching themes: Ingrained but negotiated social hierarchies; Demoralising resource and capacity deficits in government services undermining VHSNC legitimacy; Contested VHSNC intersectoral authority despite widespread intersectoral needs and responsibility; Fragmented and opaque accountability for supporting the VHSNC; Underpinning power politics; and Parallel systems.

### Ingrained but negotiated social hierarchies

Social hierarchies in Manujpur undermined some components of collective action. These hierarchies were maintained by segregation in terms of housing, access to amenities, and interaction at social gatherings. In some VHSNC trainings and meetings, caste groups sat separately and resisted sharing food, water, and tea. A lower caste woman was not told about VHSNC meetings, in what she explained was an attempt to exclude her from the committee. When she did come to meetings, she squatted beside the rug that the other VHSNC members sat upon. Higher caste people could directly fill their water vessels at the communal taps; lower caste people had to wait until the end and ask a higher caste person to fill their vessel. A lower caste woman reported that VHSNC members used the committee meeting to discuss how to better segregate water sources.

Minority groups, such as Muslim families, were derided by anganwadi workers, ASHAs, and ANMs for resisting immunization and sterilization, with exaggerated claims of fertility: “even if they [Muslims] have 10 children they don’t go for [sterilization] operation” (Jhorkibas, female, SC Hindu, AWW, IDI_VHC_30). Hindu VHSNC members sometimes blamed Muslims for not caring enough about health issues. In one instance, Hindu respondents blamed Muslims in the village for supporting the doctor who was supposed to work in their primary health center when he refused to live in the village, because the doctor was Muslim.

Social norms constrained women’s ability to participate actively in the VHSNC because they prohibited them from speaking to men in their communities and discouraged travel outside the village (where trainings and cluster level meetings were often held). Meetings generally consisted of women sitting together in silence while men spoke, with male VHSNC members sometimes unable to name the women on the committees: “since they remain behind the veil it is difficult to know every woman. And they do not speak much” (Shadeeka, male, SBC Hindu, IDI_VHC_26).

Social hierarchies also influenced community perceptions of and positionality of ASHAs, anganwadi workers, and ward members as VHSNC actors. These actors tended to be people from politically influential families, who would not speak out against one another. For example, when discussing the inadequate provision of supplementary nutrition by the Sojjanpur anganwadi, the ASHA explained: “As we belong to the same caste and same family, I cannot point fingers at them… Whatever work they wish to do, they do it. If they don’t, they don’t” (Sojjanpur, female, SC, ASHA IDI_VHC_04).

VHSNC members not related to the frontline health and nutrition workers were also constrained, since they were hesitant to speak out against dominant families. VHSNC members from lower caste or Muslim families were particularly concerned about speaking out. An SC (marginalized caste) woman in the Shadeeka VHSNC explained that in VHSNC meetings: “no one speaks because [it] would create conflict” but that her situation is particularly precarious: “That is why they don’t say anything. I have only one house of my caste. I remain afraid because everyone attacks me” (Shadeeka, female, SC, Hindu, IDI_VHC_46).

As pervasive and corrosive as social hierarchies were to community and VHSNC functioning, some social hierarchies were strategically side stepped at specific instances. Senior and respected men from each caste and religious community came together to make decisions around the “dos and don’ts of one’s clan and its tradition and rituals” (IDI, Sojjanpur, male VHC_20) or to deal with conflicts. However, while these groups worked across caste and religious boundaries, they reinforced gender norms by excluding women.

Although social hierarchies constrained broad collective dialogue, NGO staff created and enforced norms of cross-caste sharing, for example refusing higher caste requests for segregated water vessels during training sessions. They sought to include and amplify voices of lower caste people and women, and appeared to showcase non-discriminatory behavior when interacting with community members and one another. The NGO’s selection of Muslim and female facilitators, and the communities’ apparent acceptance of these facilitators, may have also subtly challenged social hierarchies. Through further mediation and leadership from the NGO facilitators, VHSNC members across caste and religious groups agreed on the key village priorities and engaged in some local action and monitoring. VHSNC meeting attendance generally included representatives from each community. Women consistently comprised the majority of VHSNC participants and despite their lower status, found avenues to be heard in the VHSNC. They would speak to their NGO facilitator about issues before or after meetings or have the most vocal female member (usually the ASHA) present an opinion for them (discussed in S1 File and [29]).

The context of social hierarchies thus permeated the VHSNCs and limited collective engagement. However these contextual features were not static and unchanging, illustrated by pre-existing cross-caste problem solving social institutions, and the VHSNC’s creation of opportunities for the careful transgressing and transformation of social norms.

### Demoralising resource and capacity deficits in government services undermining VHSNC legitimacy

While social hierarchies at the village level could be negotiated, creating space for VHSNC’s to maneuver, their legitimacy was undermined by the state of government services that they were meant to facilitate linkages with.

Government health centers were often non-functional and health worker vacancy rates were very high. The primary health centers and health sub-centers that were meant to serve our case study villages had no staff, electricity or supplies for most of the research period. A male VHSNC member in Jhorkibas explained that without basic health facilities, the entire concept of a health committee seemed illogical: “Everyone says, ‘What is a health committee if there are no facilities?’” (Jhorkibas, male, SC Hindu, IDI_VHC_44).

ANMs were to be members of the VHSNC in the village where they resided and were to serve as “special invitees” in the other VHSNCs within their catchment of five villages [20]. However, two of our case study villages did not have ANMs assigned to cover them and the existing ANMs were covering double or triple their standard catchment area because of vacancies, making it impossible for them to visit all villages regularly. Furthermore, since the health centres lacked residential quarters, ANMs commuted to the villages from the city and were too busy travelling between villages to attend meetings.

ASHAs were meant to convene the VHSNC meetings each month but two of our four villages (and 20 of the 50 intervention villages) did not have active ASHAs. In many cases the ASHAs explained that they had no training and were not seen as health workers by community members. Anganwadi centers had one or two female staff, but most lacked toilets, electricity, water, and supplies (e.g. rugs, tables, chairs), and opened only occasionally to distribute supplementary nutrition (called *panjiri*), rather than daily to provide cooked meals and preschool programmes.

As part of their convening role, ASHAs were in charge of maintaining the VHSNC registers and received Rs. 150 (US$3) for showing a signed attendance register each month. The ASHA supervisors checked the VHSNC meeting registers, but did not have time to be present for most meetings. An ASHA supervisor explained that he oversees 32 ASHAs, “And in one month, it isn’t possible to visit 32 places” (male, ASHA Supervisor, IDI_HS_07). He was already busy keeping other records about the ASHA’s work in immunization and maternal health. The ASHA supervisors had not yet been given any training about supporting the VHSNC and were not held responsible for ensuring genuine VHSNC functionality, despite this being within their remit as “the main vehicle of community mobilization and monitoring” [20].

Respondents positioned their reluctance to become involved in the VHSNC in the context of prior failed efforts to engage with government departments. For example, residents of Shadeeka submitted a complaint about the poor quality of teaching in the public school and the fact that no one was available to teach 10^th^ standard. Despite assurances from the department, their complaint never resulted in any action, apparently because no one in the village would pay a bribe to move the file forward. School management committees, an earlier government mechanism for community engagement, were reported to have been poorly supported by the Education Department, to have achieved little, had much of their budget stolen, and to have long ago stopped meeting.

Community members questioned the value of joining the VHSNC when there was so little being offered from the government and such poor experience with other government departments. An elderly man in Hanwari who was considering becoming involved in the VHSNC listed all the government failures and questioned how this activity would result in improved service provision:

There is no ANM in this village… there are no health services available in this village. The school is also a government school, and the students have to go to Manujpur [main town] and [major city] for better schooling… Even the water system here is not maintained by the government. Even roads are also not that good… I have seen that no leaders of any political parties come here… Then how will they come for this developmental activity [i.e. the VHSNC]? (Hanwari, male, ST Hindu, IDI_VHC_10)

### Contested VHSNC intersectoral authority despite widespread intersectoral needs and responsibility

VHSNCs were expected to bring together diverse actors: “The mandate of the VHSNC encompasses Health, Sanitation and Nutrition as well as the Education, particularly in the context of the programmes like Mid Day Meal, and most importantly Department of Woman and Child Development” [20]. The VHSNC is to provide “oversight and monitoring” and to “take action on social determinants of health” for the following:

- Subsidized food rations from public distribution system
- Access to work under an employment guarantee program
- Mid-day meals
- Anganwadi services
- Safe drinking water
- Access to toilets
- Female literacy
- Women and child health

These services and programs are managed by at least seven distinct departments and ministries at different levels of government. However, the VHSNC did not receive formal support or endorsement from any of them. For example, VHSNCs struggled to involve anganwadi workers, the salaried female community workers who run village pre-school and nutrition centers under the Department of Women and Child Development’ Integrated Child Development Scheme (ICDS). Anganwadi workers were expected to play a key role on the VHSNC yet were at times forbidden by their supervisors from participating in VHSNC activities. When the NGO staff asked them to attend trainings, the anganwadi workers reported that their supervisors refused to relieve them from their duties at the anganwadi center, even though anganwadi centers were frequently shut for other reasons, and told them not to show the VHSNC their record books. Respondents speculated that the anganwadi supervisors were uncomfortable with the VHSNC’s efforts to monitor anganwadi records on the distribution of nutritional supplementation, since there was corruption in the system. VHSNC efforts to work with the ICDS system and monitor the centers would have been bolstered by a clear mandate from the Department of Women and Child Development. The NGO director explained:

The problem faced at this time is that the ICDS department or health department does not provide support. Then what would VHNSC members do alone? They don’t have powers too, nor do they have support. When their supporting system is ready then progress can be seen (NGO director, female, general caste, IDI_NGO_07)

VHSNCs were positioned by the MoHFW guidelines as mechanisms for addressing small, village level issues (such as cleaning, local record keeping, local service monitoring and health education) or following up with the health system about service gaps. However, an urgent need for improved access to clean water was the single most pressing issue for all villages. The following discussion with men in Jhorkibas was typical across our research sites:

*Respondent 2*: We don’t want anything, don’t need a single rupee or tea but only do something on water and give us drinking water…

*Respondent 1*: Drinking water is a major problem for us.

*Respondent 2*: Apart from that, we don’t need anything. (men, Jhorkibas, FGD_COM_08)

VHSNCs could not engage in the large scale infrastructure projects required to address this problem, such as installing water treatment facilities, piping water from other regions, or digging new bore wells. Nonetheless water was discussed constantly in all four case study sites, at almost all the VHSNC meetings. VHSNC members in Jhorkibas and Hanwari appealed to their panchayat leaders to assist them, but explained that these requests were highly unlikely to change anything because panchayat leaders lacked resources or were solely focused on improving their own village (discussed further in “Underpinning power politics,” below). The VHSNC’s incapacity to bring about improved water access was seen to be a major indicator of the committee’s limited utility.

### Fragmented and opaque accountability for supporting the VHSNC

From the perspective of VHSNC members, there appeared to be no one in higher levels of the health administration accountable for or invested in VHSNC success.

The opacity of higher-level responsibility for supporting VHSNCs emerged most starkly around accessing the untied fund. Over the course of the 1.5 year research period, the VHSNCs never received their Rs. 10,000 yearly untied fund. Despite extensive efforts by VHSNC members and NGO staff, no one at the block level was able to identify which government actors were responsible for delivering the untied fund. They were told at different times that that the money was coming soon, that the money was not being released to the VHSNCs because the VHSNCs had not asked for it, and that the money was not being released because utilization certificates documenting expenditure could not be found from ANMs from 2007 to 2010. In 2010 responsibility for the untied fund shifted from the ANM to the ASHA, but ASHAs had not yet received the money, perhaps because the government required utilization certificates from the ANMs from previous years.

The failure of the untied fund to arrive hindered VHSNC functionality for several reasons: it constrained village action because of money shortages; it increased community skepticism of the VHSNC; and it was seen as indicative of a lack of concern from higher levels:

*Man 1*: It is the same way like in farming we put so many efforts, but if no crop comes, then what is the use of doing so much work? Similarly, when no funds have come, no development has taken place, and then everyone is of the opinion that this is useless.

*Man 2*: Nobody believes in the committee…

*Man 1*: Yes, they always say that Rs. 10,000 fund was supposed to come for development work in village. Where is the money? Why has it not come? When we say that officers above us will send the fund soon they say it is not coming; you guys are just wasting time. (Shadeeka, men, FGD_VHC_09)

VHSNC members sought accountability from block level health system actors for personnel issues and clinic management. However, the health system functionaries that VHSNC members could access (ASHA supervisors and block level medical officers) lacked the power to address most issues. At several cluster meetings, the BCMO explained to community members that he did not have the power to hire more staff and requested that communities put pressure on politicians to solve the personnel shortages:

Although I am the head of this CHC, some aspects, including the appointment of doctors, are in the hands of politicians. I am not authorized to appoint doctors. The only thing I can do is move doctors from one institution to another within the CHC area. We have to keep on demanding services so that politicians become aware of the problem and pressure the concerned ministries to solve those issues. (Observation notes, cluster meeting, OBS_VHC_26)

He explained that the Manujpur CHC was supposed to have 11 doctors but only had one, who was actually a dentist. To manage, he re-assigned three PHC doctors in the block to the CHC, but was now being pressured by the VHSNCs to return the doctors to the village PHCs. While the BCMO made it clear that he could not hire new staff, it was not clear to VHSNCs whether he was responsible for other issues, such as medicine availability, ambulance services and ANM performance. The BCMO also reprimanded the VHSNCs for not making greater efforts to address staffing issues themselves but was not clear about the mechanism through which VHSNCs were to seek political solutions to their health personnel shortages.

### Underpinning power politics: potential roles for political decentralization and media

**Fig 1:**
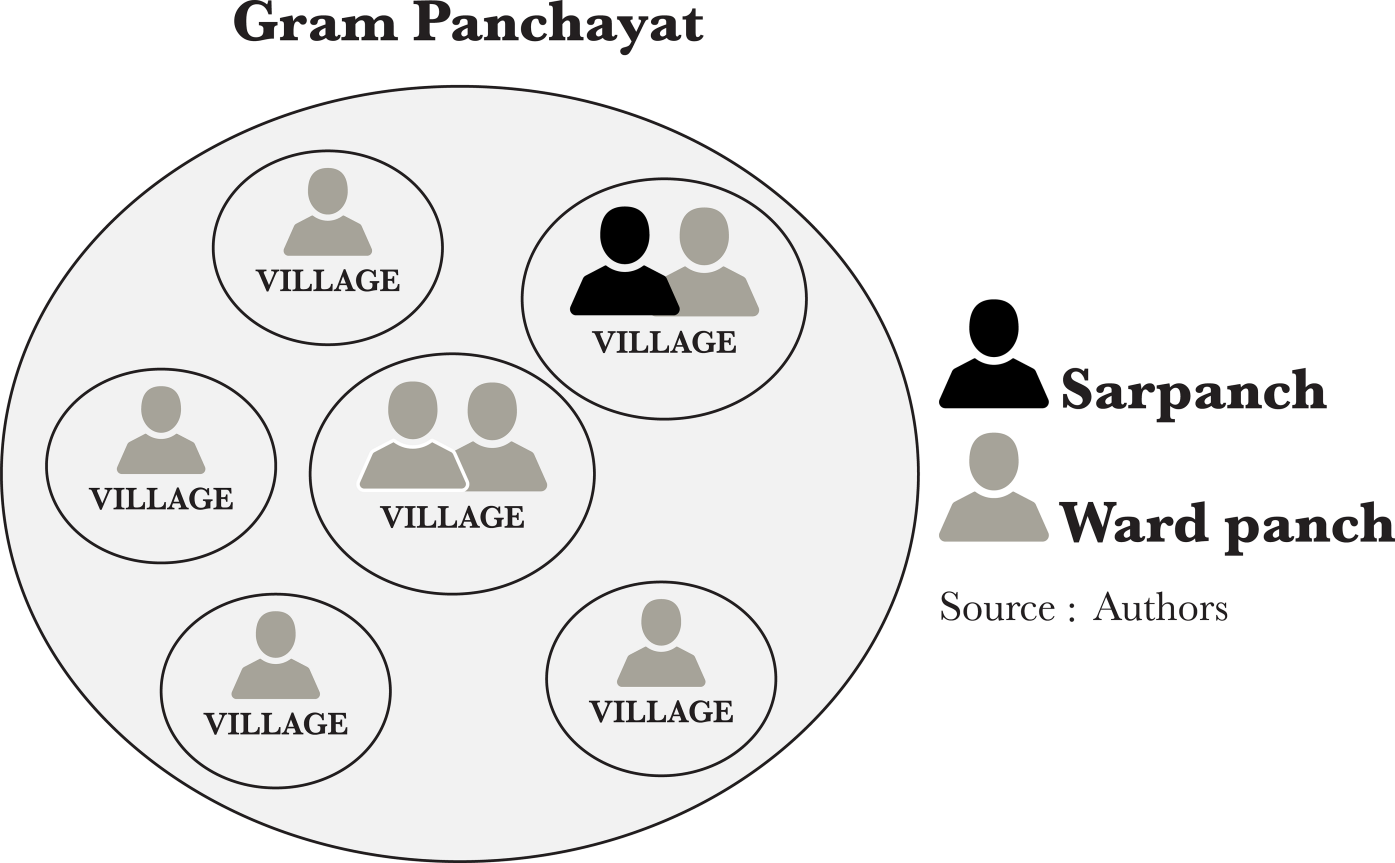
Diagram of the gram panchayat (local elected government) system.

Political decentralization and engagement with the media were both mediated by power politics, and point towards opportunities to create more enabling VHSNC contexts. Decentralization is at the heart of the Indian 73^rd^ constitutional amendment and the National Health Mission, where VHSNCs are to serve as a key mechanism for “Decentralizing Health Planning” [20]. VHSNCs were designed to access formal political authority through the gram panchayat system, by including the village’s ward panch as a VHSNC member. Each gram panchayat is led by a sarpanch and composed of between four and 10 villages, each with one or two elected ward panch (Fig 1).

However, the VHSNC members found that their linkage to the panchayat system did not enable them to mobilize funds or services to address village needs, largely because the ward panch was seen to be powerless. The sarpanch was said to funnel whatever benefits he or she could to his or her own village, such as road improvements, new wells, employment, and subsidies for building toilets, and to show little concern for the other villages in the gram panchayat. The ward members did not have their own budgets for their villages and could only request assistance for their villages from the sarpanch, which was generally denied. Most VHSNCs could not engage the sarpanch: several sarpanch outright refused to attend VHSNC meetings, citing other more pressing commitments.

While political decentralization stopped one level beyond the VHSNC’s reach, a robust local media presented an opportunity to amplify demands generated through village level collective action. While none of our four case study villages engaged with the media to try to solve their problems, another village within the intervention area, Garijwara, received assistance from local newspapers when demanding improved water. Garijwara had such poor water quality that no one would allow their daughters to marry into the village and some families were locking their homes and leaving. Women traveled 1.5 kilometers on foot to fetch water from a well that provided potable water. Although it is not possible for us to attribute the village’s action directly or exclusively to the VHSNC, we noted that the village enacted a specific plan immediately after VHSNC members attended their first training. At the training, the NGO facilitator had suggested that the VHSNC file a complaint to the sub-divisional magistrate, which it did. Shortly after filing this complaint, all the men in the village staged several day-long protests in front of the sub-divisional magistrate’s office in the nearby city.

The sarpanch, a resident of Garijwara, told the local journalists that they should highlight the village’s water issue, pointing out that he frequently buys advertisements in their newspapers. Several journalists covered the protests and visited the village. Women proudly told us that they spoke to the journalists and even allowed their names and photographs to be used, which was considered exceptionally bold. Many of the articles mentioned the VHSNC as a player in the efforts. One of the local papers covered Garijwara’s efforts for 12 straight days, with the journalist even following up himself with government officials. Community members in Garijwara felt that this ongoing coverage ultimately created enough pressure for the sub-divisional magistrate to take action. Within two weeks, a piped water system was installed in Garijwara.

Although this triumph in Garijwara occurred during the first quarter of the intervention, and was discussed at many VHSNC trainings and cluster meetings, none of the other 50 villages engaged similar media processes. The particularly stark nature of Garijwara’s plight and the Garijwara sarpanch’s clout at the local papers may have been the unique ingredients that encouraged the media to engage with this village and not others.

Political connections were important to securing benefits for one’s village or family, but often functioned through informal access to power, rather than through official democratic channels. In Hanwari, community members recalled writing an application to the collector (a government administrator) to get their subsidized food ration shop’s hours increased. They delivered it in a group of 15 people and the collector said the matter would be addressed. However no action took place, reportedly because the contractor for the ration shop was politically connected. The local political and media landscape thus suggest a system where some individuals and villages have too much political connection, while the majority have too little. Identifying strategies for VHSNCs to better leverage the democratic power of decentralized politics and media engagement offers one avenue towards increasing committee effectiveness.

### Parallel systems: the private sector and small-scale financial groups

VHSNCs operated within a context of multiple parallel systems, some of which provided counteracting incentives that undermined participation in the VHSNC.

Given the poor state of government health services, even the poorest villagers turned to market solutions when desperate, particularly for emergency health care. A few wealthier community members in our research area also turned to the market for private schooling and water (by paying to dig bore wells on their property or paying to bring water from other areas by tanker). In Hanwari, the comparatively affluent village, several respondents explained the community’s reluctance to participate in the VHSNC partially because of their ability to opt out of the public sector.

In addition to private sector options undermining collective action to improve public services, small-scale financial groups undermined community motivation to attend VHSNC meeting by creating expectations that community meetings ought to be linked to financial gain. Loan groups, also called self-help groups, were initiated bank or government representatives to help a group (primarily of women) receive a loan or pool their savings for rotating within-group lending. While loan groups imparted some bookkeeping skills that could facilitate VHSNC functioning and helped normalize the idea of women gathering for meetings outside the domestic sphere, they also conditioned community members to see meetings and committees as worthwhile when there was a clear individual financial benefit. An NGO facilitator articulated how this expectation of financial gain hindered community willingness to engage in the VHSNCs.

[Earlier] it was related to money: they used to deposit their savings and get a loan. So their association must have been due to money… No one had to be called for a meeting. They used to come on their own at the said time and place. In [the VHSNC] project we are talking about rights and there is nothing related to money… People are taking this thing negatively and people are not able to get connected with this project. (male, Muslim, IDI_NGO_04)

Nonetheless, in all the villages, including Hanwari, respondents still preferred government provision of water, schools, and sanitation since private sector solutions were expensive and piecemeal. For example, wealthier people with tractors could drive to other villages at their own expense and fetch water during droughts, but still sought a long term solution through government investment in improved water technologies and infrastructure. Desire for improved government services was unanimous, even as people expressed disappointment in the government’s failure to provide them. Therefore, community members broadly agreed with the VHSNC’s focus on strengthening the public sector, while also doubting whether this was possible and, in many cases, being reluctant to personally invest time in pressing the government to act.

## Discussion

This paper has explored the relationship between contextual features and VHSNCs in rural north India, informed by George, et al.’s framework on the influence of social, health system, administrative, and community context [8]. In doing so, it illustrates that intervention success is contingent on far more than direct technical inputs. Rather than considering the form and function of interventions in isolation, complex health system reforms must be implemented in conjunction with efforts to foster supportive contexts, and must be designed to enable responsiveness to context.

The social hierarchies that pervaded village life were both replicated and challenged in the VHSNC. Resource and capacity deficits in government services created practical challenges in building relationships between the VHSNC and frontline health workers, since so many health worker positions were vacant. More broadly, these deficits bred community scepticism that the VHSNC could bring about any positive change, particularly in light of prior failed collective attempts. Fragmented and opaque administrative accountability made it difficult for VHSNC members to follow up on requests and gain access to the authorities with the capacity to bring about change. The VHSNC’s narrow authority and mandate coupled with the villages’ widespread and inter-sectorial needs curtailed VHSNCs power. Parallel systems (political decentralization and the media) created dynamic contexts for VHSNC activity, and pointed towards untapped possibilities to engage local resources. Many people turned to the private sector for services (including emergency health care) and were accustomed to loan groups that provided direct financial benefit, which undermined motivation to participate in the VHSNC’s collective, public-oriented work.

Despite a long history of being denied government services and rebuffed by officials, most people continued to desire improved public services and some were willing to make efforts to improve them. VHSNCs were successfully formed, with membership adhering to government guidelines, and functioned over the course of the 1.5 year intervention, with trainings, regular meetings, and some local health action. This contextual analysis focuses attention beyond the internal workings of VHSNCs, to identify features of an alternative, supportive context across the four spheres identified in the original George et al. [8] framework (Table 2).

**Table 2:** VHSNC-enabling contexts.

Contextual sphere		Features of a VHSNC enabling context
Community	•	**Ongoing facilitation**: NGO facilitators played a crucial role in breaking down social hierarchies, enabling women to participate and local issues to be identified without causing village-level conflict. The NGO also worked hard to overcome community reluctance and helped VHSNCs develop strategies to appeal to the government for improved services.
	•	**Gender sensitive VHSNC support**: Ongoing strategies must support women’s participation in socially acceptable ways.
Health facilities	•	**Adequate resources**: VHSNCs need to interact with minimally functional health services; high vacancy rates make it impossible for VHSNCs to have productive engagement with health system functionaries.
	•	**Support for supporters**: Health system functionaries need training, support and incentives to work with VHSNCs.
Administration	•	**Clear pathways of accountability**: The hierarchy of responsibility for services must be conveyed to VHSNCs, so block level functionaries are not unfairly blamed for lapses beyond their control, and so the VHSNCs can engage with decision makers.
	•	**Designated top down responsibility for VHSNCs**: Make VHSNC funds and support a top-down responsibility, rather than a bottom up battle.
	•	**Intersectoral coordination**: Health administration must ensure the support of the sectors VHSNCs are mandated to engage.
Society	•	**Deep decentralization**: Decentralization to gram panchayats is a major victory for citizen engagement. However, power remained one step beyond the reach of most villages, concentrated with the sarpanch. VHSNCs would have greater scope if elected ward members had greater political and financial power and if the sarpanch was responsive to the VHSNCs. VHSNCs may be able better leverage panchayat power, perhaps through harnessing community mobilization around local elections.
	•	**Media engagement**: Develop strategies to engage media to increase VHSNC voice, enable others to learn about VHSNC actions, and generate pressure on government services to respond to VHSNC requests.
	•	**Continued public investment in the social sector:** Community participation can help guide and strengthen government health, sanitation, and nutrition programs and services, but without adequate investment, the poor will need to pay for private substitutes or suffer without basic services.

The porous nature of context and health committees, emphasized by George et al. [8], is striking. While this study identified major contextual barriers to success, these interventions and the communities themselves are not simply victims of context. Strategic renegotiation of social hierarchies, made possible by NGO support, worked to surmount some barriers at the community level (c.f. S1 File and [29]). Enabling health committees to make use of the decentralized system of local government is crucial to committee effectiveness. Similarly, committees have the potential to find allies and advocates in the media and through other local forums and groups. Some steps towards this goal can be instituted within committee form and functioning: Health committees can be designed and supported to more effectively engage the sarpanch and ward panch, perhaps through harnessing pre-existing village level coordination during the local elections, to develop media strategies, and to identify and harness the local resources created through other social institutions.

However, recognizing the committee’s capacity to influence context must not overshadow the urgent need for top down legislative changes and more coherent policy to enable and nurture local action. Leaving committees to flounder in a hostile context causes frustration and disengagement, and makes their sustainability unlikely once the NGO’s facilitation is withdrawn (c.f. [29]). The development of enabling environments [30] in which VHSNCs can not just increase community participation in trainings and meetings but actually bring about improvements in their health, sanitation, and nutrition, requires greater policy coherence from the national to block levels. Although the MoHFW’s National Health Mission policy officially promotes community participation in the public system through VHSNCs, the lower level health workers and officials lack the power, resources, and incentives to support VHSNCs and respond to many community needs. Furthermore, the inter-sectorial nature of VHSNCs reflects an ideal that must be backed up by collaboration across government ministries and departments at the higher levels of government.

VHSNCs, the National Health Mission, and entire public service sector exist in a context of public expenditure on social services far below minimum recommended levels to meet basic public needs [31,32]. In 2014, the central Ministry of Finance further reduced health expenditure [33,34]. There appears to be a fundamental discord between the MoHFW’s goals (in terms of both rapidly improving health outcomes for the poor and engaging communities) [35] and the government’s health expenditure, which remains around 1% of the GDP [32]—far below the Ministry’s goal of 2–3% [36]. The severely under-resourced public sector undermined VHSNCs in many ways, for example inviting communities to participate in a system that lacked many key staff with whom to engage. While the VHSNC is envisioned to partially fill service gaps through voluntary social service (such as working to clean the village), achieving national health goals such as reduced maternal mortality and improved child nutrition requires far more than grassroots volunteerism.

Walt and Gilson [37] argued that policy analysis has tended to focus too much on the content of reforms, rather than processes, actors and contexts. This analysis highlights the powerful effects of contextual barriers and enables us to develop an understanding of the key elements of a VHSNC enabling context. Future research must explore how to engage processes and actors to move towards this enabling context.

## Conclusions

This intervention succeeded in its core elements of improving committee form and functioning; it expanded committee membership, trained VHSNC members, facilitated monthly meetings and helped VHSNCs take local actions for health. Yet, contextual barriers played a major role in limiting VHSNC capacity to improve local health, sanitation, and nutrition. Technical inputs that build community capacity are necessary but not sufficient. In order for VHSNCs to be sustainable mechanisms for community participation in health, they need to operate within more supportive contexts that go beyond the technical inputs. VHSNC capacity to navigate and respond to contextual barriers must be harnessed, while enabling environments for health committees must be nurtured through policy and resource allocation that supports local action and genuinely invests in rural health, sanitation and nutrition.

## Supporting information

S1 File Scott, K., George, A.S., Harvey, S.A., Mondal, S., Patel, G., Raman, VR, Sheikh, K. Government helper and citizen advocate? A case study of the multiple roles and pressures facing a non-governmental organization contracted by government to strengthen community health in northern India. Status as of 29 June 2017: Unpublished manuscript, submitted to a journal for consideration.
(DOCX)

S2 File In-depth interview guide: Health system functionaries (block medical officer and ASHA supervisors). (DOCX)

S3 File In-depth interview guide: Village health committee members. (DOCX)
