# Supplementary material for: Beyond form and functioning: Understanding how contextual factors influence village health committees in northern India
Source: PLoS One. 2017 Aug 24;12(8):e0182982. doi: 10.1371/journal.pone.0182982 (PMC5570342; doi:10.1371/journal.pone.0182982)
Supplement: S1 File — A case study of the multiple roles and pressures facing a non-governmental organization contracted by government to strengthen community health in northern India. Status as of 29 June 2017: Unpublished manuscript, submitted to a journal for consideration. (DOCX) [file pone.0182982.s001.docx]

Government helper and citizen advocate? A case study of the multiple roles and pressures facing a non-governmental organization contracted by government to strengthen community health in northern India

Submitted to *Development and Change*

Status as of 29 June 2017: Submitted

**Authors**

1. Kerry Scott, PhD, Johns Hopkins School of Public Health, Baltimore, Maryland, USA & Independent Global Health Consultant, Bangalore, India
2. Asha George, DPhil, University of the Western Cape, Cape Town, South Africa
3. Steven A. Harvey, PhD, Johns Hopkins School of Public Health, Baltimore, Maryland, USA
4. Shinjini Mondal, MPH, McGill University, Montreal, Canada
5. Gupteswar Patel, MPH, The University of Newcastle, Australia
6. Raman VR, MPH, Water Aid, Delhi, India
7. Kabir Sheikh, PhD, Public Health Foundation of India, Delhi, India

1. Abstract

*Background*

Non-governmental organizations (NGOs) potentially play a valuable role in strengthening citizen engagement because of their community orientation. At the same time, the proliferation and professionalization of NGOs can contribute to a de-politicization of development, particularly when NGOs take up donor-driven projects or government contracts. Amidst these competing pressures, NGO experience and agency in managing multiple roles require further examination.

*Methods*

This paper presents a case study of an NGO implementing a government-designed health systems intervention to strengthen Village Health Sanitation and Nutrition Committees (VHSNCs). We draw from observation (n=54), interviews (n=76), focus group discussions (n=18), and secondary review of project documents. Thematic network analysis enabled the identification of themes, development of connections between these themes, and detailed exploration of sub-themes.

*Findings*

Despite a challenging context of community scepticism and poor government responsiveness, the NGO was able to successfully form and support VHSNCs over a 1.5-year period. The NGO’s respected interlocutor status in communities, pre-existing relationships, and willingness to “sell” involvement in the VHSNC as a mechanism for improving village-level health and wellbeing fostered understanding between communities and local government functionaries and enabled communities to voice concerns to government officials. However, despite these positive processes, concrete improvements were often below community expectations. NGO staff endured community frustration on one hand and rebuffs from lower-level officials on the other, while feeling under-resourced and under-supported by the contract. Consequently, while contracted to strengthen a community institution, the NGO increasingly worked alongside VHSNC members to try to strengthen the public sector more broadly.

*Conclusions*

Contrary to assumptions that NGOs become “tamed” through taking government contracts, being contracted to deliver technical inputs for community participation was intertwined with micro-level political action. While these processes enabled some positive social change, they also came at a cost to the NGO and its field staff.

*Keywords*

Non-governmental organizations; community mobilization; accountability; advocacy; empowerment; policy implementation; qualitative research; civil society; village health committees; India

2. Background

The term non-governmental organization (NGO) encompasses an enormous range of groups, from large international development institutions to small community-based collectives (Nichter, 2008). The value of NGOs in development has been attributed to two capacities: an ability to fill gaps in delivering social services to communities (“service delivery functions”) and an ability to challenge unequal relationships between government and citizens (“civil society functions”) (Banks, Hulme, & Edwards, 2015).

Over the past forty years, governments have been encouraged to shift social service provision away from the public sector and towards NGOs and markets (Banks et al., 2015; Pfeiffer, 2003). This shift was rooted in neo-liberal arguments that the public sector was inefficient and ineffective, while NGOs could provide better services at lower prices (Baqui, 2008; Fowler, 1988; Gilson, Sen, Mohammed, & Mujinja, 1994; World Bank, 2000; Zaidi, Gul, & Nishtar, 2012). The growth in funding for NGOs has led to their proliferation, as well as their professionalization and de-politicization as they moved away from civil society functions towards an increasing service delivery role (Ayers, 2006; Kamat, 2003, 2004; Miraftab, 1997; Srinivas, 2009). NGOs that arose from social movements and were once primarily volunteer-driven and politically engaged in addressing social inequalities, became increasingly “tamed” (Kaldor, 2003): staffed by inter-changeable professionals (O’Reilly, 2011), oriented toward record keeping and audits (Strathern, 2000), dependent on donors, and engaged in delivering technical services (Kamat, 2003, 2004; Miraftab, 1997; O’Reilly, 2011). Government contracts encourage NGOs to focus on implementing externally designed programs on strict timelines with quantifiable targets, reducing time for relationship-dependent empowerment activities (O’Reilly, 2011; Zaidi, Mayhew, Cleland, & Green, 2012).

Despite these trends, NGOs continue to play powerful roles in development. Some theorists argue that NGOs exhibit agency in managing risks and that many successfully retain their civil society attributes (Green, 2012). Some suggest that NGOs can play a politically charged, rather than incrementalist, role even when working as service providers. For instance, NGOs can develop a “politics of the possible” through showcasing improved alternatives to the *status quo* (Beyrer & Pizer, 2007; Nichter, 2008). Furthermore, NGOs can retain a unique capacity to develop social capital within society, especially around bridging various groups into larger collectives, linking social groups across power differentials for policy change (Nichter, 2008; Racelis, 2008; Shepard, 2006; Waghmore, 2012).

This paper examines the multiple roles and pressures that faced NGO field workers in northern India as they worked on a government contract to promote community participation in the health system. We position this case study within the broader debate about the role NGOs play in society, which questions whether providing services through government contracts limits NGO potential to effectively advocate for community needs through political engagement.

3. Methods

This paper draws from data collected as part of a larger study in two states of India on strengthening Village Health, Sanitation and Nutrition Committees (VHSNCs). Here we present one NGO’s experience over the 1.5-year period during which it implemented a government-designed VHSNC support package in a northern state (discussed further in [removed for peer review] et al.).

3.1 Study setting

The Indian government promotes the involvement of NGOs in the government health system (MoHFW India, 2013a). Five percent of the Ministry’s National Health Mission budget is earmarked for NGO contracts, which translated to Rs. 1055 crore (US $157.7 million) in 2013-2014 (MoHFW India, 2013a).

Launched in 2007, VHSNCs have been officially formed in over 500,000 villages across India and are a key part of the government’s strategy to engage rural communities (MoHFW India, 2005, 2013b). These committees are to meet each month, develop village health plans, take action to improve local health, nutrition, and sanitation, monitor the performance of local health and nutrition services, and allocate a yearly “untied fund” of Rs. 10,000 (US $150). Membership brings together the village’s female community health worker, called the accredited social health activist (ASHA), the anganwadi worker (AWW, a female pre-school and nutrition worker), elected ward representative in local government (called the panchayat system), and other interested community members, such as members of savings collectives and parent-teacher associations.

**The VHSNC-support intervention**: The NGO, called SEEK (a pseudonym), implemented the MoHFW’s VHSNC support package in 50 villages, covering a rural population of approximately 68,000 people. The support package consisted of social mobilization in each village to increase community understanding of and support for VHSNCs, expanding VHSNC membership from seven to 15 members, training the VHSNC members, and supporting their monthly meetings and activities. After getting the committees up and running, quarterly cluster level meetings were also convened, which brought together two or three members from each VHSNC in a cluster of 16-17 VHSNCs.

**Profile of the NGO**: SEEK was founded in the early 2000s by a now-deceased Indian public figure. SEEK had a central office in the state capital and several field offices in highly marginalized locations across the state, including the field office in a region called Manujpur (a pseudonym), where this study took place. SEEK’s work focused on educational programs, such as running village libraries and summer schools. They primarily received funding from private trusts and corporate social responsibility initiatives. SEEK’s work on this VHSNC-strengthening intervention was their first health project.

Of the 50 villages receiving the VHSNC-strengthening intervention, SEEK had previously run development projects in 31 villages. The depth of SEEK’s involvement in these villages was moderate: SEEK ran small libraries or short camps rather than any large programs or infrastructure projects. Only some village residents knew of SEEK and the VHSNC-support intervention was run by new field staff, hired specifically for the VHSNC intervention.

The SEEK field office in Manujpur, which had been set up in 2008, was a simple five-room cement house located at the edge of Manujpur town (population 11,000). The seven SEEK staff who implemented the VHSNC-intervention were primarily from Manujpur or nearby blocks and included three women and four men. They all had previous development work experience and were of mixed social backgrounds, including a Muslim and Hindus from “other backwards castes” (OBCs; a marginalized caste group) and general castes (non-marginalized)—although none were scheduled caste (SC) or scheduled tribe (ST), the two most marginalized caste groups.

**Social overview of the region**: According to the 2011 Indian census, there were about 200 villages in Manujpur Block, with an average population of 1300 per village. The population was approximately 17% SC and 12% ST. While male literacy was reported at 80%, female literacy was only 50% and had not improved since the 2001 census. Based on its own data collection, SEEK estimated the Muslim population at over 20%, with literacy among Muslim women as low as 13%. Most residents were poor farmers who raised buffalo and grew mustard, wheat, and sorghum on small plots of land or worked as migrant laborers in farming, construction, or driving.

The region was poor and resource-deprived. Roads were potholed and nearly impassible during monsoons; schools, anganwadi centers, and health facilities were crumbling; toilets were rare; and water and electricity were in short supply. The public health system was extremely understaffed. According to government norms, one auxiliary nurse midwife (ANM) should serve approximately five villages, but in this region, ANMs covered 15 villages or more. Several primary health centres (PHCs) had no staff or operated with one or two staff members when there should have been at least 13.

3.2 Study design

Throughout SEEK’s implementation of the VHSNC support package, a team of researchers conducted in-depth implementation research over 1.5 years (2014-215). This paper draws from in-depth interviews (IDIs) with SEEK’s staff, VHSNC members, non-VHSNC community members, and health system actors; focus group discussions (FGDs) with community members; ongoing observation and informal discussion with NGO staff; as well as secondary review of written project materials (table 1).

[table 1 here]

Most of the data collection was conducted by XX, a 25-year-old male Hindi-speaking researcher who lived in Manujpur during the study, closely supported by the study’s research associate (YY) and research coordinator (ZZ), as well as the broader study team of senior researchers. The interviews and focus group discussions were conducted in Hindi and translated and transcribed into English. Each transcript was carefully checked by XX or YY, and then approved by ZZ. Key words were retained in Hindi and the team returned to and discussed the original Hindi audio to clarify particularly nuanced sections. Observation notes were written in English.

3.3 Data analysis

Our analysis was guided by thematic network analysis (Attride-Stirling, 2001), whereby a coding framework was developed, based on our *a priori* theoretical interest in mechanisms that influence community participation through VHSNCs and ameliorated by codes that emerged from the data itself, including the challenges faced by the NGO facilitators. This coding framework was applied to the text, supported by the qualitative software ATLAS.ti, to enable us to group text segments by code. We then read and re-read the data outputs as they were presented for each code and extracted the salient, common, and significant basic themes, which were then clustered into larger organizing themes. The organizing themes relevant to the NGO’s experience implementing this intervention are presented as the sub sections in the findings section: (1) Navigating a challenging context to implement the intervention; (2) NGO characteristics and strategies to increase the intervention’s success; (3) Combining service delivery with civil society engagement; and (4) Challenges and costs born by SEEK as a result of implementing this intervention.

4. Results

4.1 Navigating a challenging context to implement the intervention

SEEK encountered community hesitancy and poor responsiveness from government providers and officials (discussed further in XX et al, forthcoming). Community responses to the intervention were colored by a widespread sense that the government was inaccessible, unfair, and uncaring. While some communities responded positively to the idea of forming a village health committee and welcomed SEEK staff, in other cases people recounted past government failures and sounded weary of investing energy in a new initiative after previous disappointments. In several instances, efforts to mobilize communities to participate in the VHSNC were met with resistance and scepticism, at times bordering on hostility:

There was restlessness among the people. They were not willing to sit patiently and listen to me… They were complaining that even after they have raised their concern, no one listens to them. They said that you or this committee cannot be of any use to us. (NGO facilitator, IDI_NGO_01)

SEEK adhered to MoHFW-issued VHSNC guidelines, which promoted VHSNCs as bodies that could monitor and coordinate local health, nutrition, and sanitation services. However, the VHSNC’s mandate came from the MoHFW without official support from any other ministries or departments, including nutrition and sanitation. An NGO facilitator explained their struggle to reconcile this theorized ideal of intersectoral collaboration with reality:

It is like the government is running away from us and we want to work closely with the government… For VHSNCs there are six services at the village level, like anganwadi, ANM, mid-day meal [in schools], etc., that should reach people. Our aim is to make people aware and demand… The twist is that people have to seek these services from six separate departments, like health, education, water, whereas the project is only with the health department. (NGO manager, IDI_NGO_02)

Several key determinants of VHSNC success lay beyond the control of the NGO. The untied fund of Rs. 10,000 (US $150) for each VHSNC failed to materialize over the course of the intervention, despite efforts by VHSNCs and SEEK staff to determine why the money was being withheld. SEEK staff also tried, unsuccessfully, to secure formal government orders supporting anganwadi collaboration with VHSNCs. SEEK staff asked officials in the health department to speak to the Integrated Child Development Services (ICDS), which oversees the anganwadi system, but despite several requests there was no evidence that this inter-departmental communication occurred. Over the course of the intervention, officials in the panchayat, health, education, and many other departments frequently failed to reply to VHSNC requests or promised improvements that never materialized.

4.2 NGO characteristics and strategies to increase the intervention’s success

Despite these challenges, SEEK succeeded in forming and training VHSNCs across the 50 villages, and was able to facilitate monthly meetings in most villages throughout the intervention period. Their support enabled female VHSNC members to participate and brought together community members from different caste and religious groups to seek improved health, nutrition and water services. In this section we describe the characteristics and strategies used by SEEK to achieve key intervention objectives.

- NGO staff motivation

Several SEEK staff spoke about the importance of community action in the public health system and expressed dedication to seeing VHSNCs strengthened: “And SEEK is working to make the [public] system better… We want people to accept the system, work with it, understand it and own it” (Project director, IDI_NGO_07). Over our observation period, the NGO staff consistently displayed high levels of commitment to seeing VHSNCs become functioning local institutions for community participation. They expended energy and time above and beyond the basic requirements to try to engage communities, train VHSNC members, and help the VHSNC improve village level health, sanitation, and nutrition services.

- Respected interlocutor status

As neither village residents nor government officials, NGO facilitators presented VHSNC members an opportunity to engage with a unique set of interlocutors. The NGO facilitators were in many ways local: they understood and identified with members of the community; people knew their family, caste, religion, and village. Yet they were outsiders who brought new knowledge and had connections to government. Unlike government officials, they did not require formal interactions and were not seen to be consumed by the indifference and self-interest often associated with officials.

In most villages, it was risky to openly discuss problems in frontline service provision, such as an anganwadi worker taking the supplementary food rations home with her (discussed further in XX et al, forthcoming). The positions of panchayat ward member, ASHA, and anganwadi worker were occupied by dominant local families, and these actors were often related to one another. For example, the ASHA and anganwadi worker were often sisters-in-law. It was difficult for relatives who occupied positions of power to criticize one another, and for lower status community members to criticize members of dominant families. Yet VHSNC members, including the ASHA, were expected to participate in monitoring the anganwadi’s work.

To overcome this challenge, VHSNC members often took SEEK facilitators into confidence about local issues, away from the larger group. Speaking to the NGO facilitator was a mechanism for taking some action and was less dangerous than reporting on one’s peers to a government worker. Sometimes NGO facilitators were then able to help address the problems. For example, SEEK facilitators sometimes stayed with VHSNC members to monitor the anganwadi center immediately after the VHSNC meeting, instead of asking the VHSNC members to conduct the monitoring exercise on their own between meetings. Although the anganwadi centers did not appear to improve as a result of this NGO-supported monitoring during our research period, the NGO’s involvement nonetheless enabled the shortcomings of the anganwadi center to be noted, and exposed anganwadi staff and community members to a set of standards.

Women, who were generally not able to speak in VHSNC meetings because of social norms prohibiting them from speaking in front of male relatives, found it socially acceptable to speak to the NGO facilitators, male and female alike. Women often had side conversations with the NGO facilitator that enabled the facilitator to present women’s concerns and suggestions to the men at VHSNC meetings. For example, a female VHSNC member explained, to general agreement, “We do not speak there [in VHSNC meetings] but speak quite a lot to the one who comes, Roopa [female NGO facilitator, all names are pseudonyms]” (FGD_VHC_11).

In some cases, the NGO facilitator would use his or her outsider status to press the female VHSNC members to speak at the VHSNC meetings in the presence of men, overlooking the local prohibition. While this demand may have made the women uncomfortable, the women usually discussed amongst themselves and then had the ASHA present their opinion, which the male VHSNC members seemed to accept.

VHSNC members also noted that visits from outsiders elevated the VHSNC meetings above a normal community activity, which helped sustain interest. For example a male VHSNC member (IDI_VHC_50) explained that the NGO facilitator “makes things lively.” If no outside facilitator came, he and many others said, no one in the village would gather. Some community members felt that information from outsiders was given greater weight and was more socially acceptable than if it came from a village resident.

- Leveraging relationships and reputation to meet program goals

NGO staff described leveraging personal and professional relationships to meet the VHSNC program’s goals. Some NGO staff had prior work relationships with teachers and anganwadi workers. SEEK staff drew upon these relationships of trust or obligation to strengthen the VHSNC. Involving actors from across departments without official documents of support depended entirely upon personal relationships. For example, NGO staff navigated the ICDS’s lack of formal support for VHSNCs by encouraging anganwadi workers to attend trainings on their personal time:

Then we said, ‘What if they come on off-duty hours?’ So some of the anganwadi workers volunteered to come and engage in training on off-duty hours. They came because of our personal and organizational relationships. (NGO manager, IDI_NGO_02)

NGO staff also leveraged prior relationships with community members to elicit VHSNC support. NGO facilitators managed community scepticism by offering up their own assurance and character as a testament to their sincerity and the positive potential of the VHSNC. Facilitators thus staked their own reputation on the intervention’s success. They promised that they would see the intervention through and that they would be available for the community:

I promised them that I will come every month and also whenever they call me. I told them that I will stay in the village for a long tenure and will continue to come even after forming the committee. The villagers said let’s see how many times you visit the village. I told them to trust me and I shared my phone number with them. (NGO facilitator, IDI_NGO_04)

- Emphasis on positive potential of intervention

Their motivation to see the intervention succeed led NGO staff to “sell” the VHSNC to communities, by emphasizing the committee’s positive potential. NGO staff presented the VHSNC as a means of getting the government to deliver on public health entitlements and allocating the yearly Rs. 10,000 (US $150) untied fund. Although NGO staff told communities that VHSNC members would have to take initiative themselves and did not explicitly promise any public health system improvements, interviews with community members indicated that many people took away a message that the NGO would bring a great amount of positive change. For instance, a woman recalled that the NGO facilitator called them during social mobilization and said that the NGO “will make all the [health] facilities available” (FGD_COM_03).

4.3 Combining service delivery with civil society engagement

In leveraging their reputations and interlocutor status, SEEK combined the finite elements of delivering contracted technical inputs with the more fluid relational civil society engagement. They did so by enabling community voice, building government capacity, facilitating mutual understanding, and supporting VHSNC linkages to external resources and actors, as well as bridges to other peer VHSNCs.

- Enabling community voice

Although the focus of their contract was to form, train, and support VHSNCs, SEEK staff began orienting many VHSNC meetings around strategies to engage government officials and advocate for local needs. To this end, SEEK staff were able to facilitate community access to government actors in ways that had previously not been possible. SEEK staff acquired the contact information for block level government officials, explained to VHSNC members which departments and officials were responsible for delivering which services, coached VHSNC members on how to speak to government agents, and accompanied VHSNC members to deliver requests. VHSNC members valued this support:

Once I went with Aasif [NGO facilitator] to our MO [medical officer]… We went to him regarding our request to provide training for the ASHA. We also went for our Rs. 10,000 untied fund… So the MO asked why we did not come earlier, [if you had] your work would be done. So one day we went directly to [the city] hospital with the letter. So Aasif helps us a lot. (Male VHSNC member, IDI_VHC_37)

NGO staff also led the process of helping VHSNCs write request letters to the government. SEEK designed, printed and distributed official looking letter pads to each VHSNC and taught members how to compose request letters and where to submit them. In meetings, almost every issue discussed concluded with the act of writing a request to the government to address a village level problem. For example, in one cluster meeting (OBS_VHC_35) we documented seven issues in which the NGO staff supported writing letters.

- Capacity building of government providers and officials

Building community capacity was an explicit component of SEEK’s mandate. But the NGO staff recognized that health system actors and members of local government also needed training, since few were aware of the VHSNC or knew the committee’s mandate. SEEK staff organized special trainings to teach newly elected local (panchayat) representatives about the VHSNC. SEEK staff also invited ASHA supervisors, ANMs, and medical officers to VHSNC trainings and meetings, and tried to build their support and interest in responding to these committees.

Previously, VHSNC members could only access the medical officers in their governmental or clinical spaces. SEEK brought these health functionaries to community-oriented spaces, such as the SEEK office, and enabled interaction with VHSNCs that took place according to the NGO’s agenda. SEEK staff exhibited careful diplomacy in treating health system actors as respected guests, while also engaging them in exercises of public accountability. Health system staff were expected to answer community questions, for example about why VHSNC funding had not yet been released, why ASHAs had not been trained or equipped with drug kits, and whether vacant positions for ANMs and doctors would be filled. While their answers were sometimes vague and rarely led to immediate tangible improvements, this dialogue between VHSNC members and government officials was nonetheless a first step towards improved accountability.

- Facilitate understanding between health system and community

The NGO’s status between community and government created opportunities for the community to understand some of the challenges facing health workers, thereby shifting some anger at individual government workers towards action at an administrative level. For instance, NGO staff explained to VHSNC members in a village with an empty health center that the doctor who should serve them had not abandoned his post but had instead been re-allocated.

Health system staff also worked through SEEK to try to build community understanding of their issues, and to engage the community in productive ways. At a cluster meeting at the SEEK office, the block chief medical officer (BCMO) explained to community members that he did not have the power to hire more staff for their health center and requested that communities put pressure on politicians to solve the staffing issue (observation notes, OBS_VHC_26).

- Linking to external resources/ actors and bridging with other VHSNC

In addition to building community-government relationships, SEEK staff also linked communities to resources and support outside the public sector, through their knowledge of other foundations and charities in the region. For example, SEEK staff connected VHSNCs to a private foundation working in the area, which expressed interest in funding VHSNC sanitation and health work, and to a local corporate social responsibility initiative working on the government’s mid-day meal program in schools.

Through trainings and cluster-level meetings SEEK created bridges between villages, enabling VHSNC members to meet and exchange ideas. The facilitators themselves served as bridges by moving between villages and bringing with them comparative perspectives. For example, in one VHSNC meeting where members protested that the committee was useless because the government was unresponsive, a SEEK facilitator told the story of a nearby VHSNC’s successful work in improving teacher performance at a school. In highlighting success stories, SEEK was able to encourage VHSNC members and help them develop locally feasible action strategies.

4.4 Unforeseen risks for NGO capacity and relationships

Despite these important civil society efforts, the positive potential of VHSNCs became problematic for NGO staff when important aspects of the intervention did not go as hoped.

- NGO became the face of the program but lacked control over many aspects

Repeated failures led to disappointment for the VHSNC members and resulted in frustration, which was frequently directed at the NGO staff. For example, some people questioned the honesty of NGO facilitators, who had said that the untied fund would be arriving in their accounts soon:

Most of the committees have been there for one year. We told them that within two months they would get the untied fund as per the information given to us. So now the difficulty is coming as they are saying that you are lying to us… We ourselves don’t have the answer about why it is not coming. (Project director, IDI_NGO_07)

While communities disparaged the government for failing to provide services, over the course of the intervention they began directing frustration at the NGO facilitators for breaking what they had understood to be a promise for improvements in the village. “People say that she speaks too much and we go mad. She doesn’t do any work related to development but only makes discussions” (VHSNC member, IDI_VHC_47).

The NGO facilitators never had any control over the construction of facilities or hiring health workers, and were themselves frequently rebuffed by block level health system actors. Their mandate was merely to form VHSNCs, train members, and help VHSNC members conduct meetings. They had no control over how the rest of the system engaged with VHSNCs, yet found themselves desperately, and generally unsuccessfully, trying to facilitate positive government engagement with VHSNCs, which would in turn justify the NGO’s promotion of these committees.

- Efforts to sustain community trust in the NGO may have reinforced community scepticism about improving government services

We observed the NGO staff engage several strategies to maintain their reputation or regain community trust in response to the barriers that VHSNCs faced. SEEK staff often spoke to one another in front of VHSNC members about the government’s rejection of the NGO’s efforts. For example, the director expressed her frustration about the BCMO and ASHA supervisor in front of about 40 VHSNC members at a cluster meeting, highlighting that the BCMO and ASHA supervisor gave conflicting explanations for why they could not attend:

*NGO director (female)*: See, this is how these government people are supporting us. They don’t want to come because they will have to answer to the public. (Cluster meeting, OBS_VHC_34)

These public actions showcased the fact that the NGO was unable (rather than unwilling) to bring about improvements, and illustrated the NGO’s community-aligned (rather than government-aligned) identity.

SEEK also sought to clarify and even downplay their role, by presenting their organization as a modest facilitator to the more important community-government relationship. For instance, VHSNC members angrily asked SEEK staff why the VHSNC had not received any money and what the VHSNC was supposed to do when the government was unresponsive. Some NGO staff responded by aggressively reminding VHSNC members that the issue is between the communities and the government:

*VHSNC member*: From the beginning you are talking about the untied fund but to date you have not given a single rupee.

*NGO manager*: This is your VHSNC and your money. So who should try to get the money?

*VHSNC member*: But you are saying that that money will come, so you are responsible for giving that money.

*NGO project director*: We are not providing the money and it is not in our hands. The money should come from the government. This is a matter between the government and VHSNC members. We are just a medium in between and we are trying to help you. (OBS_VHC_35)

In showing the VHSNC members that SEEK was not at fault, the NGO both encouraged and discouraged sustained community participation in the public sector. SEEK encouraged participation and ownership by emphasizing that communities had the right to demand their entitlements directly (without SEEK acting as an intermediary). SEEK facilitators even suggested the VHSNCs file a Right To Information request to find out where the money was. However, some of their efforts to sustain their community-oriented identity may have adversely affected community willingness to continue engaging in the committees. Emphasizing the challenges SEEK faced when trying to work with government made some VHSNC members wonder how the VHSNC could succeed if even SEEK staff were themselves unsuccessful.

In writing petitions to the government on every issue, SEEK staff took the only clear action available to them and aligned themselves as community advocates. The act of writing petitions to the government at the end of meetings satisfied the short-term expectation from VHSNC members to see the NGO facilitator take some action. Nonetheless, these acts of writing achieved near-symbolic status, becoming a representation of action, rather than meaningful action itself. The main outcome and action of SEEK staff, and as the intervention was phased out, of VHSNC members themselves, was the creation of letters. With minimal government response to these letters, the short term satisfaction of “doing something” generally led to a longer term reinforcement of the government’s inaccessibility and the VHSNC’s ineffectiveness at resolving upstream public system issues.

- NGO staff felt their work was under-valued and under supported

NGO staff were concerned that the government-designed intervention underestimated the time and resources required for field activities. Properly introducing the concept of VHSNCs to entire villages, gaining community trust, and successfully convening meetings required more visits, and at multiple times of day, than facilitators were financially supported to undertake or felt possible in the time allotted:

Say you have to arrange a VHC meeting in one day. But arranging meetings requires time and mobilization. And it is possible that we need to go more than once…. The government needs to understand that they have to give priority to people. We work like that only. The government says work 10 am to 5 pm and finish everything. It becomes very rigid. (NGO manager, IDI_NGO_02)

Female facilitators were in a particularly difficult situation. They could not make use of the NGO-owned motorcycles, because it is unacceptable for women in this region to drive motorcycles. They had to walk or arrange to be driven by men—public transportation was not available to the villages.

Another facilitator reported feeling overwhelmed by a hostile crowd during a mobilization event and suggested that additional staff would have helped him: “It is not possible for a single person to handle so many people. Support could be in terms of additional staff to gather and counsel people” (IDI_NGO_01). However, SEEK often lacked the time and human resources for facilitators to work in pairs.

In addition, NGO staff and VHSNC members were concerned that VHSNCs would not continue to function once “handed back to the government” with NGO support removed.

According to me if the project doesn’t continue then there won’t be any meetings or any other activities. It would become non-functional like before…. Now that we repeatedly visit and persuade them so much we have been able to motivate them a little. It won’t be possible in the future if they are not given any back up support. (NGO facilitator, IDI_NGO_05)

The NGO director also pointed out that the end of the project was a major challenge for their own organization’s development. SEEK did not have enough money after the VHSNC-support intervention closed to retain their staff:

I am seeing a challenge too that now this project is getting over… The passion and enthusiasm that is in them now… we don’t have resources [to sustain] that… So I am feeling that perhaps it should not stop here. A lot of energy and manpower has been invested in this project… At this point of time they are emerging as trainers and if we leave those team members right now then our investment on manpower would be lost. (Project director, IDI_NGO_07)

As predicted, SEEK was unable to keep the employment contracts of several staff who worked on the VHSNC initiative.

5. Discussion

This paper illustrates the challenges, trade-offs, and strategies enacted by an NGO implementing a government-designed health system intervention. The NGO worked within a difficult context of community mistrust and poor government responsiveness to successfully form and support VHSNCs over a 1.5-year period. Their involvement brought together community representatives to discuss local issues, and enabled some monitoring of local government services, and opportunities for women’s voices to be heard. They helped communities voice their concerns to government officials, and to facilitate some understanding between communities and local level government functionaries. They built the capacity of some government officials to work with VHSNCs and helped link communities with one another and with other sources of non-governmental support. This was possible due to the NGO’s respected interlocutor status in communities, pre-existing relationships, and willingness to “sell” involvement in the VHSNC as a mechanism for improving village-level health.

Despite enabling some positive processes, concrete improvements in the villages were often below community expectations. Government support for the intervention was considered inadequate by NGO staff and communities, particularly evidenced by the government’s failure to release untied funds into VHSNC accounts, as well as poor cross-departmental collaboration and minimal response to VHSNC requests for improved services. NGO staff thus endured community frustration on one hand and rebuffs from lower-level government officials on the other, while feeling under-supported by their government contract. The NGO risked its reputation in communities by promoting VHSNCs without being able to control crucial aspects of the intervention’s success. While the NGO became a conduit for government messages and values, it did not gain any of the power associated with government bureaucracy, such as access to district-level government actors or information on disbursement of funds.

Sustaining community participation in the VHSNC was contingent on community members seeing the VHSNC as a mechanism to improve local wellbeing, which required upstream political action. Thus, fulfilling contractual requirements focused on changing community members (i.e. building capacity and willingness to participate) was intertwined with taking up a civil society oriented role that worked on changing the system (e.g. engaging government officials, encouraging communities to demand entitlements). Facilitating small-scale political action enabled SEEK to maintain the trust and participation of the community members. Despite concern that government contracts lead to de-politicization (Banks & Hulme, 2012; Kamat, 2003, 2004; Miraftab, 1997; Zaidi, Mayhew, et al., 2012), our findings show that providing the technical services required by some government contracts necessitated micro-level political engagement. Whether this micro-level action can contribute meaningfully to broader political change remains a fundamental question (Banks et al., 2015), but nevertheless suggests that an NGO’s civil society capacity can be mobilized by service delivery contracts.

This case study also illustrates that, as noted by others (George & Branchini, 2017; O’Reilly, 2011; Zaidi, Mayhew, et al., 2012), NGOs take significant risks through engaging in these contracts. This is especially true when formal government promotion of community participation (exhibited by the existence of these contracts and national policies) does not translate to ground reality (exhibited by weak government capacity to engage with communities). NGOs must navigate power relations which strain community trust and can lead to distressing interactions with frustrated community members (Cohen-Blankshtain, Ron, & Perez, 2013). The intervention ended with many communities unconvinced about the VHSNCs’ utility. This does not bode well for sustained community engagement in VHSNCs and may make it more challenging to work with these communities on future initiatives. Moreover, after all their work to strengthen VHSNCs, many of the SEEK facilitators lost their jobs at the end of the contract, emphasizing the high individual and organizational costs of NGO ‘professionalization’ in response to short-term contracts (O’Reilly, 2011).

NGOs can contribute both a service delivery role and a rights-based civil society role to community development (George & Branchini, 2017). They bring important characteristics and strategies born of their position between communities and governments. However, the nature of these contractual relationships must be carefully considered (Palmer, 2000): NGOs need adequate time, resources, and support from the government to strengthen community-government relationships (Shepard, 2006; Zaidi, Mayhew, et al., 2012). They must proceed with caution to protect their organizational interests and ensure that short-term technical work serves longer-term social goals.

We note several limitations and opportunities for future research. First, the presence of researchers throughout this intervention’s 1.5-year implementation period may have influenced NGO staff behavior. For instance, it could have encouraged SEEK staff to exert more effort during their work or influenced how critical they were of their experience with the government contract. However, the extended and embedded nature of this research is likely to have tempered this reactivity. Second, we note several perspectives that require analysis. Future research is needed to examine how government policymakers and bureaucrats view the role of NGOs in service delivery and community empowerment, including social accountability initiatives. While we interviewed government health workers at the block and village level, we did not interview higher-level government stakeholders for this study. In addition, research is required among NGO directors to understand how their organizations are responding to government contracts, in terms of long-term strategies, structures, and priorities. While our case study details how one field team worked within the bounds of a specific contract, research among NGO leadership would shed light on how specific implementation experiences influence long-term engagement with government and communities.

6. Conclusions

NGOs have a valuable role to play in increasing community participation in government systems. As interlocutors, they fill a unique position in society that enables them to strengthen community-government relationships. Frontline NGO staff can support communities to assert their rights to public services. This engagement is contrary to assumptions that NGOs are necessarily depoliticized and “tamed” by development contracts. However, NGOs must recognize the risks and trade offs associated with trying to maintain an identity as community advocate while serving as a government contractor. The risks are particularly acute in contexts where the government system lacks the incentives and capacities to effectively respond to newly engaged community members.

**8. Works cited**

Attride-Stirling, J. (2001). Thematic Networks: an analytic tool for qualitative research. *Qualitative Research*, *1*(3), 385–405.

Ayers, A. J. (2006). Demystifying democratisation: the global constitution of (neo)liberal polities in Africa. *Third World Quarterly*, *27*(2), 321–338.

Banks, N., & Hulme, D. (2012). *The role of NGOs and civil society in development and poverty reduction* (BWPI Working Paper Series No. 171). Manchester. Retrieved from http://www.bwpi.manchester.ac.uk/medialibrary/publications/working_papers/bwpi-wp-17112.pdf

Banks, N., Hulme, D., & Edwards, M. (2015). NGOs, States, and Donors Revisited: Still Too Close for Comfort? *World Development*, *66*, 707–718.

Baqui, A. H. (2008). NGO facilitation of a government community-based maternal and neonatal health programme in rural India: improvements in equity. *Health Policy and Planning*, *23*(4), 234–243.

Beyrer, C., & Pizer, H. (2007). *Public Health and Human Rights: Evidence-Based Approaches*. Baltimore: Johns Hopkins University Press.

Cohen-Blankshtain, G., Ron, A., & Perez, A. G. (2013). When an NGO Takes on Public Participation: Preparing a Plan for a Neighborhood in East Jerusalem. *International Journal of Urban and Regional Research*, *37*(1), 61–77.

Fowler, A. (1988). *Non-Governmental Organizations in Africa: Achieving Comparative Advantage in Relief and Micro-Development* (No. Discussion Paper 249).

George, A. S., & Branchini, C. (2017, forthcoming). Principles and processes behind promoting awareness of rights for quality maternal care services: A synthesis of stakeholder experiences and implementation factors. *BMC Pregnancy and Childbirth*.

Gilson, L., Sen, P. D., Mohammed, S., & Mujinja, P. (1994). The potential of health sector non-governmental organizations: policy options. *Health Policy and Planning*, *9*(1), 14–24.

Green, D. (2012). What can we learn from a really annoying paper on NGOs and development? Retrieved October 22, 2015, from http://oxfamblogs.org/fp2p/what-can-we-learn-from-a-really-annoying-paper-on-ngos-and-development/

Kaldor, M. (2003). The idea of global civil society. *International Affairs*, *79*(3), 583–593.

Kamat, S. (2003). The NGO Phenomenon and Political Culture in the Third World. *Development*, *46*(1), 88–93.

Kamat, S. (2004). The privatization of public interest: theorizing NGO discourse in a neoliberal era. *Review of International Political Economy*, *11*(1), 155–176.

Miraftab, F. (1997). Flirting with the enemy: challenges faced by NGOs in development and empowerment. *Habitat International*, *21*(4), 361–375.

MoHFW India. (2005). *National Rural Health Mission: Mission Document*. New Delhi.

MoHFW India. (2013a). *Financial Outlays & Outcome Budget 2013-14, Chapter 2*.

MoHFW India. (2013b). *Guidelines for Community Processes*. New Delhi.

Nichter, M. (2008). NGOs, Social Capital, and the Politics of the Possible. In *Global Health: Why Cultural Perceptions, Social Representations, and Biopolitics Matter* (pp. 133–149).

O’Reilly, K. (2011). “We Are Not Contractors”: Professionalizing the Interactive Service Work of NGOs in Rajasthan, India. *Economic Geography*, *87*(2), 207–226.

Palmer, N. (2000). The use of private-sector contracts for primary health care : theory , evidence and lessons for low-income and middle-income countries. *Bulletin of the World Health Organisation*, 821–829.

Pfeiffer, J. (2003). International NGOs and primary health care in Mozambique: The need for a new model of collaboration. *Social Science & Medicine*, *56*(4), 725–738.

Racelis, M. (2008). Anxieties and affirmations: NGO–donor partnerships for social transformation. In A. Bebbington, S. Hickey, & D. Mitlin (Eds.), *Can NGOs make a difference? The challenge of development alternatives* (pp. 196–218). London and New York: Zed Books.

Shepard, B. (2006). *Running the obstacle course to sexual and reproductive health: lessons from Latin America*. Westport, Connecticut: Praeger.

Srinivas, N. (2009). Against NGOs? A critical perspective on nongovernmental action. *Nonprofit and Volunatary Sector Quarterly*, *38*(4), 614–626.

Strathern, M. (2000). *Audit Cultures: Anthropological Studies in Accountability, Ethics and the Academy*. Routledge.

Waghmore, S. (2012). Beyond Depoliticization? Caste, NGOs and Dalit Land Rights in Maharashtra, India. *Development and Change*, *43*(6), 1313–1336.

World Bank. (2000). *The World Bank-civil society relations: Fiscal 1999 progress report.* Washington DC.

Zaidi, S., Gul, X., & Nishtar, N. (2012). Parallel NGO Networks for HIV Control: Risks and Opportunities for NGO Contracting. *Global Journal of Health Science*, *5*(2), 171–176.

Zaidi, S., Mayhew, S. H., Cleland, J., & Green, A. T. (2012). Context matters in NGO-government contracting for health service delivery: a case study from Pakistan. *Health Policy and Planning*, *27*(7), 570–81.
