# Supplementary material for: Beyond form and functioning: Understanding how contextual factors influence village health committees in northern India
Source: PLoS One. 2017 Aug 24;12(8):e0182982. doi: 10.1371/journal.pone.0182982 (PMC5570342; doi:10.1371/journal.pone.0182982)
Supplement: S2 File — (DOCX) [file pone.0182982.s002.docx]

**In-depth interview guide: health system functionaries (Block Medical Officer and ASHA supervisors)**

| Domain/topic | Questions and probes |
| --- | --- |
| 1. Identity and role in health system | Can you please tell me a bit about your work as BMO/ASHA supervisor?   - What are your key tasks? - How did you become BMO/ASHA facilitator? - What do you enjoy about the work? - What do you find challenging? |
| 2. Purpose and role of village health committee (VHC) | Can you tell me, from your perspective, what is the purpose of the VHC? What activities should the VHC do?  What should the membership be like? |
| 3. Exposure to VHC | When did you first hear about the VHC? What did you think about it?  Have you ever discussed the VHC with other people, such as your colleagues? What did you talk about? |
| 4. VHC formation, inclusiveness and representativeness | Do you know about how the committee was formed and how members got selected?   - What do you think about this process? Was it fair? What could have made the VHC formation better?   What people should be VHC members? Why?  Does the VHC membership truly represent the community?   - Are there communities or key people that are not represented on the VHC?   Who is most active on the VHC? Who is least active? What are some reasons for this? |
| 5. VHC embeddedness in the community | What is the relationship between the general community and the VHC?   - To what extent are VHC members aware of the issues facing the community? - What do you think the community expects from the VHC? - Do you think the VHC is meeting the expectations of community members? |
| 6. Relationship between VHC and health services | Have you ever interacted with VHC members? What was the interaction about? What happened?  How does the health system respond to and interact with the VHC?   - Does anyone from the health system come to VHC meetings or activities? Who? What do they do/say? - If the VHC wants to change something about the health system, what can they do? How would the health system respond? Why? Possible probes:   ANM coming regularly  108 services/Mobile Medical Unit  Primary Health Centre open longer  Doctor more regular  Medicines more available  RKS/JSY payments  Anganwadi services   - Tell me about the relationship between health workers (ANM/anganwadi) and VHC members   Have you ever heard about the VHC taking some sort of health action or planning role, such as sending requests to the health department? Tell me about what happened. What do you think about this? |
| 7. VHC activities and functionality | Have you ever heard about the VHC monitoring health facilities, immunization, VHNs or anganwadi facilities? What do you think about this? Describe how this monitoring works. What are some challenges? What is done with this monitoring data? How do you feel about this monitoring activity?   - What issues do you think the VHC should work on? - What things do you think the VHC can actually do? - Has the VHC or any VHC member spoken with community members about health? Has the VHC taught people about the health system? How?   Record keeping   - Does the VHC maintain any registers? Keep minutes? Who does this? Do these records help? |
| 8. Meetings and health planning | - Have you ever been involved in VHC meetings? Tell me about the meeting - Where would it take place? What do you think about this location? - Who would be there? Who would not attend the meeting? - What kinds of things are talked about? (Have you ever talked about violence, particularly against women?) - What makes you happy to go to the meetings? What makes you not want to go? - Who mostly talks? Who mostly stays quiet? Why? - Do the meetings address the real issues? - Have you heard of the VHC doing any health planning activities (Village Health Plan/Panchayat Health Plan/Primary Health Centre health plan)? Tell me about this process. |
| 9. VHC training | Have you ever been taught about the VHC in any meetings or trainings? If yes, tell me about this. If no, what would you like to learn or know about the VHC? |
| 10. Untied fund | Please tell me about your experiences with the untied fund?   - What has been done to try to get it released? - How is the lack of untied fund affecting the VHC functioning? - If the untied fund came what would you do with it? |
| 9. Changes over time related to VHC | Have you seen any changes come out because of the VHC?   - The VHN/ANMs visits to the community? Her behavior? - Water availability and cleanliness? - The Anganwadi center and anganwadi worker? Whether the rations are given, whether the center is open at the correct times, whether children use the center, whether their weight is monitored? - The extent to which very marginalized people are accessing health services? - The ASHA’s work, knowledge, support? - People’s awareness of the VHC? Of their health rights? - The availability of care and quality of care from the health centers? Availability of drugs? Staff absenteeism? 108? - Village Health and Nutrition Days/activities? - Whether people use public health services versus private health services? - Receiving cash incentives for delivery? - Children being vaccinated?   If the respondent notes a change, ask them why they think this change has occurred.  Whenever the respondent says nothing has changed, explain “the past year, there have been efforts to strengthen the VHC so that it could help improve things. Yet ____ has not improved. What might be some reasons for this lack of improvement?” |
| 10. VHC hopes and concerns | - What do you think the VHC will do over the next year? - Do you think the VHC will continue functioning without [NGO’s] help? Why/why not? - What are the best things about the VHC? What are your hopes for the VHC? - What are the challenges facing the VHC? What would help the VHC overcome these challenges? - **What do you think needs to change to make the VHC more functional? Probe on:**   - How would different aspects (other members, resources, training, health system, other stakeholders) need to change? |
