# Supplementary material for: Beyond form and functioning: Understanding how contextual factors influence village health committees in northern India
Source: PLoS One. 2017 Aug 24;12(8):e0182982. doi: 10.1371/journal.pone.0182982 (PMC5570342; doi:10.1371/journal.pone.0182982)
Supplement: S3 File — (DOCX) [file pone.0182982.s003.docx]

**In-depth interview guide: village health committee (VHC) member**

*Note: different sections were emphasized at different times during the research and according to the respondent’s identity; some section were skipped entirely so that sufficient time would be available for sections that required deeper discussion. For example, during the early months of the intervention we focused on VHC formation and member training, and did not discuss health system responsiveness since the VHCs were only at the formation and training stage.*

| **Domain/topic** | **Questions and probes (optional; use as needed)** |
| --- | --- |
| 1. Identity and VHC involvement | I’m interviewing you to learn as much as I can about VHCs. Can you tell me about how you are involved in the VHC?   - What is your role? - What do people at home (husband/wife/in-laws/child) think about your being on the VHC? - How much time each month do you spend on VHC activities? Is this okay? - What are the good things about being a member? What are the bad things? - Can you tell me about a time when you were happy to be a member? Can you tell me about a time when it was not easy being a member? |
| 2. VHC formation | How was the committee formed?   - How did you become a member? - How were the other members selected? What do you think about this process? Was it fair? What could have made the VHC formation better? |
| 33. VHC inclusiveness and representativeness | Does the VHC membership truly represent the community?   - Who are the members of the VHC? What communities are they from? Are there representatives from women’s groups? - Are there communities that are not represented on the VHC? (ST/SC/OBC, other minorities) - Who is most active on the VHC? Who is least active? What are some reasons for this? |
| 4. VHC embeddedness in the community | What is the relationship between the general community and the VHC?   - What do community members think about the VHC? - Tell me about the relationship between community members and VHC members. What about the most marginalized people? - Tell me about a time when a VHC member told the community something about the VHC. Has there ever been a time when community members were suspicious about what the VHC was doing? Have VHC members ever felt that the community did not understand them? - In the past year has anyone in the village asked you about the VHC? What did they ask? - In the past year, did you ever tell anyone in the village about the VHC? - To what extent are VHC members aware of the issues facing the community? - What do you think the community expects from the VHC? - Do you think the VHC is meeting the expectations of community members? |
| 5. Relationship between VHC and health services | How does the health system respond to and interact with the VHC?   - Does anyone from the health system come to VHC meetings or activities? Who? What do they do/say? - If the VHC wants to change something about the health system, what can they do? How would the health system respond? Why? Probe:   ANM/VHN coming regularly  Primary Health Centre open longer  Doctor more regular  Medicines more available  RKS/JSY payments  Anganwadi services   - Tell me about the relationship between health workers (ANM/anganwadi) and VHC members |
| 6. VHC activities and functionality | Activities   - Can you/any member tell me about a time when the VHC tried to solve a problem? - Can you/ any member tell me about a time when someone came to the VHC for help? - What issues do you think the VHC should work on? - What things do you think the VHC can actually do? - Has the VHC or any VHC member spoken with community members about health? Has the VHC taught people about the health system? How? - Has the VHC monitored any services (AWC, quality of care or availability of care at health centers, immunization, water tank, 108, etc…)? Describe how this monitoring works. What are some challenges? What is done with this monitoring data? How do you feel about this monitoring activity?   Record keeping   - Does the VHC maintain any registers? Keep minutes? Who does this? Do these records help? |
| 7. Meetings and health planning | Can you describe a typical committee meeting?   - Where would it take place? What do you think about this location? - How regularly does the VHC meet? Who calls people for the meetings? - Who would be there? Who would not attend the meeting? - Who coordinates/leads the meetings? Who keeps minutes? - What kinds of things are talked about? (Have you ever talked about violence, particularly against women?) - What makes you happy to go to the meetings? What makes you not want to go? - Who mostly talks? Who mostly stays quiet? Why? - Do the meetings address the real issues? - Tell me about how VHC members get along with one another (men & women, people from different communities, health system representatives)? Has there ever been an argument? What happened? - Have you ever been involved in health planning activities (Village Health Plan/Panchayat Health Plan/PHC health plan)? Tell me about this process. |
| 8. VHC training | Can you tell be about the trainings that the committee received?   - What were some of the things you learned about? - What did you like about them? What did you not like? - Which people in the VHC were unable to attend the training? Why was this? How could the training be changed so that more VHC members are able to attend?   [If the interviewee did not attend ask about why: Why did you miss the training? What could have been changed to make it easier for you to attend? Did you hear about the training from other people? What did they tell you?] |
| 8. Untied fund | What do you know about the untied fund?   - What has been done to try to get it released? - How is the lack of untied fund affecting the VHC functioning? - If the untied fund came what would you do with it? |
| 9. Changes over time related to VHC | Over the past year, have you noticed any changes to:   - The ANMs visits to the community? Her behavior? - Water availability and cleanliness? - The Anganwadi center and anganwadi worker? Whether the rations are given, whether the center is open at the correct times, whether children use the center, whether their weight is monitored? - The extent to which very marginalized people are accessing health services? - The ASHA’s work, knowledge, support? - People’s awareness of the VHC? Of their health rights? - The availability of care and quality of care from the health centers? Availability of drugs? Staff absenteeism? 108? - Village Health and Nutrition Days/activities? - Whether people use public health services versus private health services? - Receiving cash incentives for delivery? - Children being vaccinated?   If the respondent notes a change, ask them why they think this change has occurred.  Whenever the respondent says nothing has changed, explain “the past year, there have been efforts to strengthen the VHC so that it could help improve things. Yet ____ has not improved. What might be some reasons for this lack of improvement?” |
| 10. VHC hopes and concerns | - What do you think the VHC will do over the next year? - Do you think the VHC will continue functioning without [NGO’s] help? Why/why not? - What are the best things about the VHC? What are your hopes for the VHC? - What are the challenges facing the VHC? What would help the VHC overcome these challenges? - What do you think needs to change to make the VHC more functional? Probe on:   - How would different aspects (other members, resources, training, health system, other stakeholders) need to change? |
